# SLC11A2: a promising biomarker and therapeutic target in ovarian cancer

**DOI:** 10.1038/s41598-022-26789-5

**Published:** 2023-01-20

**Authors:** Liming Tian, Xuemei Li, Huiling Lai, Tingting Sun, Xiaohui Li, Linxiang Wu, Chuling Wu, Shuzhong Yao, Yufeng Ren, Shasha He, Guofen Yang

**Affiliations:** 1 Department of Gynecology, The First Affiliated Hospital, Sun Yat-Sen University, No. 58, Zhongshan Road II, Guangzhou, 510080 China; 2 Department of Gynecology, Qilu Hospital of Shandong University (Qingdao), Jinan, China; 3 Department of Laboratory Medicine, The First Affiliated Hospital of Xiamen University, Xiamen Key Laboratory of Genetic Testing, School of Medicine, Xiamen University, Xiamen, 361005 China; 4 Department of Gynecology, The Sixth Affiliated Hospital, Sun Yat-Sen University, Guangzhou, China; 5 Department of Radiotherapy, The First Affiliated Hospital, Sun Yat-Sen University, Guangzhou, China

**Keywords:** Ovarian cancer, Diagnostic markers, Prognostic markers

## Abstract

Ovarian cancer has the highest mortality rate among gynecologic tumors, with a 5-year survival rate of less than 25%. There is an urgent need for early diagnosis and new drugs to reduce the disease burden of ovarian cancer. The aim of this study was to investigate the effectiveness of SLC11A2 as a therapeutic target and marker for ovarian cancer. Expression data of SLC11A2 were obtained from public databases. Then, the biological functions of SLC11A2 were validated in four ovarian cancer cell lines. Finally, we collected ovarian cancer clinical tissues, serum, and plasma exosomes and used immunohistochemistry, Elisa, and liquid chromatography-mass spectrometry (LC–MS) to validate the test efficacy of SLC11A2. The results showed that ovarian cancers with high SLC11A2 mRNA expression had shorter 5-year PFS and MST. Knockdown of SLC11A2 reduced ovarian cancer migration and increased cisplatin-induced apoptosis. Serum SLC11A2 may help improve the detection rate of ovarian cancer.

## Introduction

Ovarian cancer in mortality ranks first among female reproductive malignancies^1, 2^. Although the use of targeted drugs such as Poly ADP-ribose polymerase (PARP) inhibitors significantly prolongs the survival time of ovarian cancer patients^3^, especially those with BRCA mutations^4^, the five-year survival rate is less than 25% to 30%^5, 6^. With the promotion of HPV vaccine^7^, cervical cytology, and HPV testing, cervical cancer can be easily prevented and screened. But ovarian cancer is more likely to spread due to its hidden onset and special anatomical location^8^, making the mortality rate the highest. Finding new molecules to provide early diagnosis and targeted therapy is the key to breaking through the predicament.

Ion channels are involved in cellular information transmission and material transport and are associated with malignant behavior in cancer^9^. In 1997, SLC11A2, an 11 member of the solute carrier family 2, was identified as an iron transporter by Gunshin et al^10^. SLC11A2 protein transports ferrous ions from the intestinal lumen to the body in a transferrin (TF)-independent manner. This is the only way the gut absorbs ionic iron from food. The carcinogenesis of iron ions has a broader research basis, but as a key molecule in iron transport, the role of SLC11A2 in malignant tumors remains unclear. By analyzing TCGA data, Yin Weijiao et al. found that SLC11A2 was associated with the prognosis of endometrial cancer^11^. A study by Kaja Michalczyk et al. showed that genetic polymorphisms in SLC11A2 were not associated with endometrial cancer risk^12^. Currently, non-bioinformatics-based superiority cancer studies have only been reported in colon and breast cancers^13, 14^.

Given that sustained iron stimulation promotes ovarian carcinogenesis^15^, SLC11A2 is a major protein for iron uptake^16^ and has been shown to play a definitive role in breast and colon cancer progression, we performed a comprehensive analysis using numerous publicly available expression and survival databases. Meanwhile, we validated the effect of SLC11A2 on ovarian cancer cells in vitro. Finally, the protein expression of SLC11A2 in serum, ovarian cancer tissue, normal ovary, and normal fallopian tube tissue, and protein expression level in the serum of ovarian cancer patients were detected.

## Methods

### Transcriptional data acquisition in pan-cancer

We examined differences in SLC11A2 expression between cancer and its normal tissues by 3 independent bioinformatic databases (Oncomine, https://www.oncomine.org/resource/login.html; GEPIA2, Gene Expression Profile Interactive Analysis 2, https://gepia2.cancer-pku.cn; GENT, Gene Expression in Normal and Tumor Tissue database, http://gent2.appex.kr/gent2/). In the GENT database, two microarray platforms (GPL570 and GPL96) were used to display the expression of pan-cancer. All databases were carried out with default settings.

### Changes of SLC11A2 genome, transcriptome, and protein in ovarian cancer

The expression of SLC11A2 mRNA and the gene mutation in the ovary and normal counterparts was analyzed with the CBioPortal database (https://www.cbioportal.org) and TCGA (https://tcga-data.nci.nih.gov/tcga/). The RNAseq data in TCGA and GTEx TPM format is unified with the TOIL process^17^. Extract TCGA's OV (ovarian serous cystadenocarcinoma) and normal tissue data corresponding to GTEx(Genotype-Tissue Expression Project; http://commonfund.nih.gov/GTEx/). R(ggplot2) was used to visualize the data. We used the UALCAN (http://ualcan.path.uab.edu/cgi-bin/ualcan-res-prot.pl) database to calculate the detection efficacy of tissue SLC11A2 mRNA for ovarian cancer. The expression of SLC11A2 protein in ovarian cancer and normal ovarian tissues was acquired in the immunohistochemical images from the Human Protein Atlas database (https://www.proteinatlas.org/). Section staining was scored using the integral system, and the staining intensity score was multiplied by the percentage score to obtain the integral value. The staining intensity score was defined as follows: 0, negative; 1, weak; 2, moderate; 3, strong. Percentage scores represent the proportion of positive cells to all cancer cells. The percentage score was defined as 1, 0–25%; 2, 26–50%; 3, 51–75%; 4, 75–100%.

### Clinical correlation between SLC11A2 mRNA and ovarian cancer

SLC11A2 expression and prognosis in ovarian cancer were investigated by the Kaplan-Mayer plotter (http://kmplot.com/analysis/) web-based tool. Four independent SLC11A2 mRNA probes were used to calculate SLC11A2 expression in each risk group. Survival curves were generated for all patient or clinicopathological data using cut-off values corresponding to the default optimal P value in the database.

The clinicopathological significance of SLC11A2 in ovarian cancer was comprehensively evaluated through the UALCAN database (http://ualcan.path.uab.edu/cgi-bin/ualcan-res-prot.pl). Clinical subgroups included FIGO clinical stage, ethnicity, TP53 mutation, age, pathological differentiation, and grade.

### Analysis of SLC11A2 Co-expression genes, signaling pathways, and gene ontology

We obtained the co-expression of ovarian serous cystadenocarcinoma with SLC11A2 with RNAseq data from the TCGA OV (Ovarian serous cystadenocarcinoma) project genes. After data cleaning, we mapped the bubble chart of the top 10 genes positively associated with SLC11A2. Subsequently, we performed gene ontology and signaling pathway analysis using the top 100 genes co-expressed with SLC11A2 in ovarian cystadenocarcinoma above. Contents include Biological Process, Kinase Class, Protein Function, Subcellular Location, Drug, Canonical Pathways, and Hallmark Gene Sets. Instead of categorical specific presentations, we used the Metascape tool (https://metascape.org/gp/index.html) to show the top 20 of all clusters.

### Determination of the effect of SLC11A2 on ovarian cancer clone formation

We overexpressed or knocked down SLC11A2 in OVCAR8 (an ovarian cancer cell line), and verified its manipulation efficiency by qPCR or Western blot. In western blot, cellular proteins were transferred to the PVDF membrane, which was then cropped into two strips according to the molecular weight of the protein marker. Primary antibody to SLC11A2 (72kD) and β-actin (42kD) was used to incubate these two strips separately. Original western blots are presented in Supplementary Fig. S1. The overexpression plasmid and negative control plasmid were designed and synthesized by Yuanjing Biotechnology (Guangzhou, China). The knockdown si-RNA and negative control si-RNA were designed and synthesized by Qingke Biotechnology (Guangzhou, China). OVCAR8 cells were cultured in a 10 cm dish, and the digested cells were divided into groups when the confluence reached 80%. The overexpression plasmid (OE), negative plasmid (NC or WT), siRNA (KD), and negative siRNA (NC or WT) were transfected respectively. 24 h after transfection, cells were re-digested, counted, and plated, at 1000 cells per well. After 10–14 days of culturing, they were fixed with formalin, stained with crystal violet, dried, and photographed. ImageJ software was used to calculate the area of colony formation and R was used to count and visualize the data. All cell function experiments were repeated at least 3 times with 3 triplicates.

SLC11A2 Western blot Primary antibody: Abclonal (Wuhan, China; catalog: A10231).

siRNA target sequence: GGAGGAATCTTGGTCCTTA, GTACCTGCATTCTGCCTTA, GAGTGACTTTGCCAATGGA.

qPCR Primer sequence: F, ATCGGCTCAGACATGCAAGAA; R: TTCCGCAAGCCATATTTGTCC.

### Determination of the effect of SLC11A2 on ovarian cancer proliferation and migration

We used siRNA to knock down SLC11A2 in ovarian cancer cell lines ES2, A2780, and SKOV3 (si-RNA group). The control group used si-RNA without a knockdown effect (NC group). Five replicate wells were set in each group, and the initial density was 1000 cells per well (96 wells plate). On the first and fifth days of culture, CCK8 was added and incubated for 2 h to detect the absorbance at 450 nm. To simulate the circumstances of clinical tumor chemotherapy, we also provided a cisplatin intervention group (CIS) to obtain whether SLC11A2 might affect platinum-based chemotherapy.

Transwell experiments were used to examine the effect of SLC11A2 on the migratory ability of ES2, A2780, and SKOV3. After digestion, the cells were added to the upper wells of the Transwell chamber in a serum-free medium, and the serum-containing medium was added to the lower wells. After 24 h of culture, the cells were fixed with methanol, washed with PBS, and stained with crystal violet; the cells in the upper layer that did not pass through the small holes were wiped off, air-dried, and photographed. ImageJ software analyzed the cell area of cells passing through the well. All cell function experiments were repeated at least 3 times with 3 triplicates.

### Explore the effect of SLC11A2 on apoptosis of ovarian cancer cells

Platinum is the basic drug for first-line chemotherapy of ovarian cancer. We cultured ovarian cancer cell lines transfected with SLC11A2-siRNA and NC-siRNA, filled 12-well plates, and added cisplatin to induce apoptosis to mimic the chemotherapy process in vivo. After 5 days in culture, cells were digested and washed with PBS. Apoptosis was detected by flow cytometry with Annexin V-FITC/PI Apoptosis Detection Kit. The 3 flow cytograms in the first row were the ‘NC’ group (Negative control siRNA), and the 3 flow cytograms in the second row were the ‘si’ group (SLC11A2 Knock down siRNA). The total amount of apoptosis is early apoptosis plus late apoptosis (two quadrants on the right). Percent apoptosis figures are shown in blue above and red below, respectively. Apoptosis experiments were repeated at least 3 times with 3 triplicates.

Annexin V-FITC/PI Apoptosis Detection Kit: AAT Bioquest (CatLog: 20092).

### Immunohistochemical analysis of SLC11A2

We collected voluntary patient specimen tissues from 2019 to 2021 in gynecological surgery patients at the First Affiliated Hospital of Sun Yat-sen University. Paraffin-embedded tissue specimens included 6 normal ovarian tissues, 6 normal fallopian tube tissues, 8 primary ovarian high-grade serous carcinomas, and 3 omentum high-grade serous carcinoma metastases. The tissue samples were fixed, embedded in paraffin, sectioned, deparaffinized, hydrated, antigen retrieved, incubated with primary and secondary antibodies, and sealed after color development. 2 slices from each group were selected and presented in the article, using 2 × and 10 × for each slice.

To verify the correlation between the expression of SLC11A2 and the prognosis of chemotherapy, we retrospectively studied the pathological sections of ovarian cancer primary lesions of patients treated in the First Affiliated Hospital of Sun Yat-sen University from 2014 to 2020. Inclusion criteria: the pathological type is high-grade serous carcinoma, FIGO stage III-IV, and the treatment method needs to include neoadjuvant chemotherapy; exclusion criteria: the primary tumor cannot be found under the microscope, combined with other malignant tumors, combined with other diseases that seriously affect life expectancy, did not complete the full course of cytoreductive surgery and postoperative chemotherapy, lost to follow-up.

According to the above conditions, a total of 68 cases with valid data were screened, and SLC11A2 immunohistochemical staining was performed on the pathological sections of primary ovarian cancer. Section staining was scored using a scoring system as described above. Immunohistochemical scores were graded independently by two persons.

SLC11A2 IHC Primary antibody: Abcam (catalog: ab262715).

### Explore the serum concentration of SLC11A2 in ovarian cancer patients

We collected 27 pre-treatment plasma samples from 9 healthy women, 9 patients with benign ovarian lesions, and 9 patients with ovarian malignant tumors in the First Affiliated Hospital of Sun Yat-sen University in 2018, respectively. All human blood samples were collected after obtaining approval from the Institutional Review Board of the First Affiliated Hospital of Sun Yat-sen University and informed consent from all participants. Whole blood samples were obtained from fasted participants using vacuum blood collection tubes. The collected plasma was centrifuged at 3000 rpm for 10 min at 4 °C, followed by 12,000×*g* for 15 min. Store at − 80 °C for backup. Batch transfer the supernatant to a new centrifuge tube and filter with a 0.22 μm microporous membrane. After protein extraction, the peptides were digested into abortions using trypsin. Generated MS/MS data were processed using the MaxQuant search engine (v.1.5.2.8) (Max Planck Institute for Biochemistry, Munich, Germany). Tandem mass spectra were searched against the SwissProt human database linked to the reverse decoy database.

Then, we collected sera from ovarian tumors and healthy female volunteers at the First Affiliated Hospital of Sun Yat-sen University and the First Affiliated Hospital of Xiamen University in 2020–2021. There were 33 samples in the control group, including healthy women, patients with post-operative ovarian cancer, patients with borderline ovarian tumors, patients with benign ovarian lesions, and patients with colon cancer. A total of 48 samples were included in the experimental group, including patients with untreated ovarian cancer and patients with confirmed recurrent ovarian cancer.

Serum SLC11A2 concentrations were measured using the SLC11A2 Elisa Avidin kit. Use the MyCurveFit web tool (https://mycurvefit.com/) to draw the Elisa standard fit curve and calculate the curve formula. The measured protein concentrations were combined with the clinical diagnosis to obtain receiver operating characteristic (ROC) curves.

ELISA Kit for Solute Carrier Family 11 Member 2: EIAAB Science Inc, Wuhan (catalog: E0316h).

Software: R (version 3.6.3) (statistical analysis and visualization);

R package: pROC package [1.17.0.1] (for analysis), ggplot2 package [3.3.3] (For visualization).

### Ethics approval and consent to participate

All projects obtained approval from respective ethics committees. The Ethics Committee of the First Affiliated Hospital of Sun Yat-sen University (ethics approval NO. 308-2016-03-01, NO483-2021-6-28). All specimens were obtained with patient consent and authorization. All methods were carried out by relevant guidelines and regulations.

## Results

### SLC11A2 is highly expressed in multiple cancers

**Figure 1:**
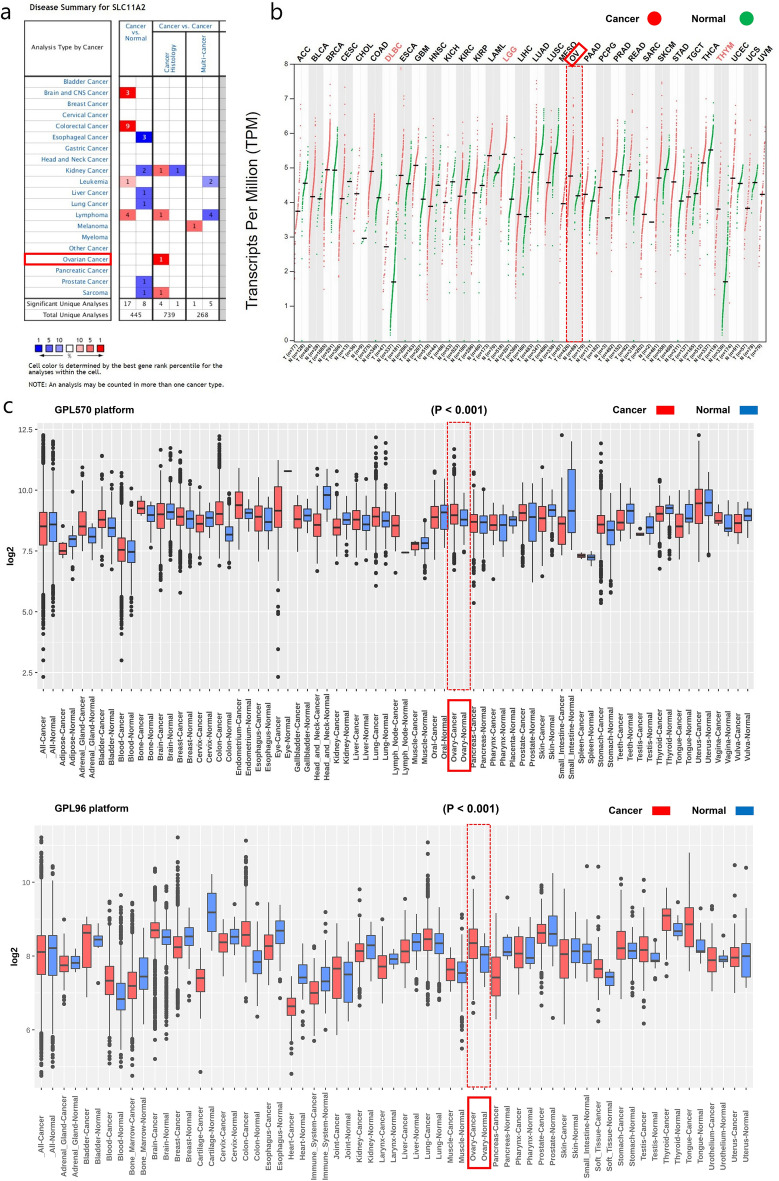
SLC11A2 mRNA expression in various cancer types. (**a**) Comparison shows the number of datasets with SLC11A2 mRNA overexpression (left column, red) and under-expression (right column, blue) in cancer compared to normal tissue. This graphical presentation is derived from the Oncomine database, and the thresholds are designed with the following parameters: p-value 1E−4, fold change 2, and gene rank 10%. (**b**) Expression of SLC11A2 in 33 human cancers by GEPIA2: gene expression profiles of all tumor samples and paired normal tissues displayed as a dot plot. Each point represents the expression of the sample. (**c**) Expression pattern of SLC11A2 mRNA in tumor and corresponding normal tissues: retrieved from the GENT2 on the expression of SLC11A2 mRNA in various cancers. Boxes represent the median and the 25th and 75th percentiles. Dots represent outliers. The red box represents tumor tissue and the green box represents normal tissue. Statistical methods: two-sample T-test.

The Oncomine database contains data from studies from multiple sources. In Fig. 1a, red squares represent high transcript levels for that gene in that cancer (p < 0.0001), and blue ones each represent low transcript levels for that gene in that cancer (p < 0.0001). The numbers in the boxes represent the number of studies supporting the trend. The Oncomine database shows that SLC11A2 mRNA was expressed at high levels in the brain and central nervous system tumors, lymphoma, colon cancer, and leukemia compared to normal tissues. Compared with cancer tissues, SLC11A2 mRNA expression was elevated in ovarian cancer tissues. (Fig. 1a). We further analyzed SLC11A2 expression between 33 human cancers and their normal tissues using expression data retrieved from the pooled TCGA and GTEx data using the GEPIA2 tool (Fig. 1b). In the GENT database, SLC11A2 expression was upregulated in several cancer types (Fig. 1c), including adrenal, bladder, bone, breast, endometrial, colon, lung, lymphoma, prostate, gastric, and ovarian cancer. Data from two independent GENT microarray platforms showed that SLC11A2 transcript levels were significantly increased in ovarian cancer compared with normal ovarian tissue (GPL570, P < 0.001, Log2FC = 0.207; GPL96, P < 0.001, Log2FC = 0.399). The results show that the expression of SLC11A2 is significantly increased in various cancer types compared to normal tissue. The expression level of SLC11A2 mRNA in ovarian cancer tissues was higher in the 3 databases (Fig. 1, noted by red boxes). Setting p < 0.001 as the threshold, the two GENT microarray data were intersected to obtain SLC11A2 mRNA with elevated transcript levels in blood, breast, colon, liver, lung, and ovary cancers and reduced transcript levels in kidney cancer. (Supplementary Table S1a,b.).

### SLC11A2 mRNA and protein are highly expressed in ovarian cancer

**Figure 2:**
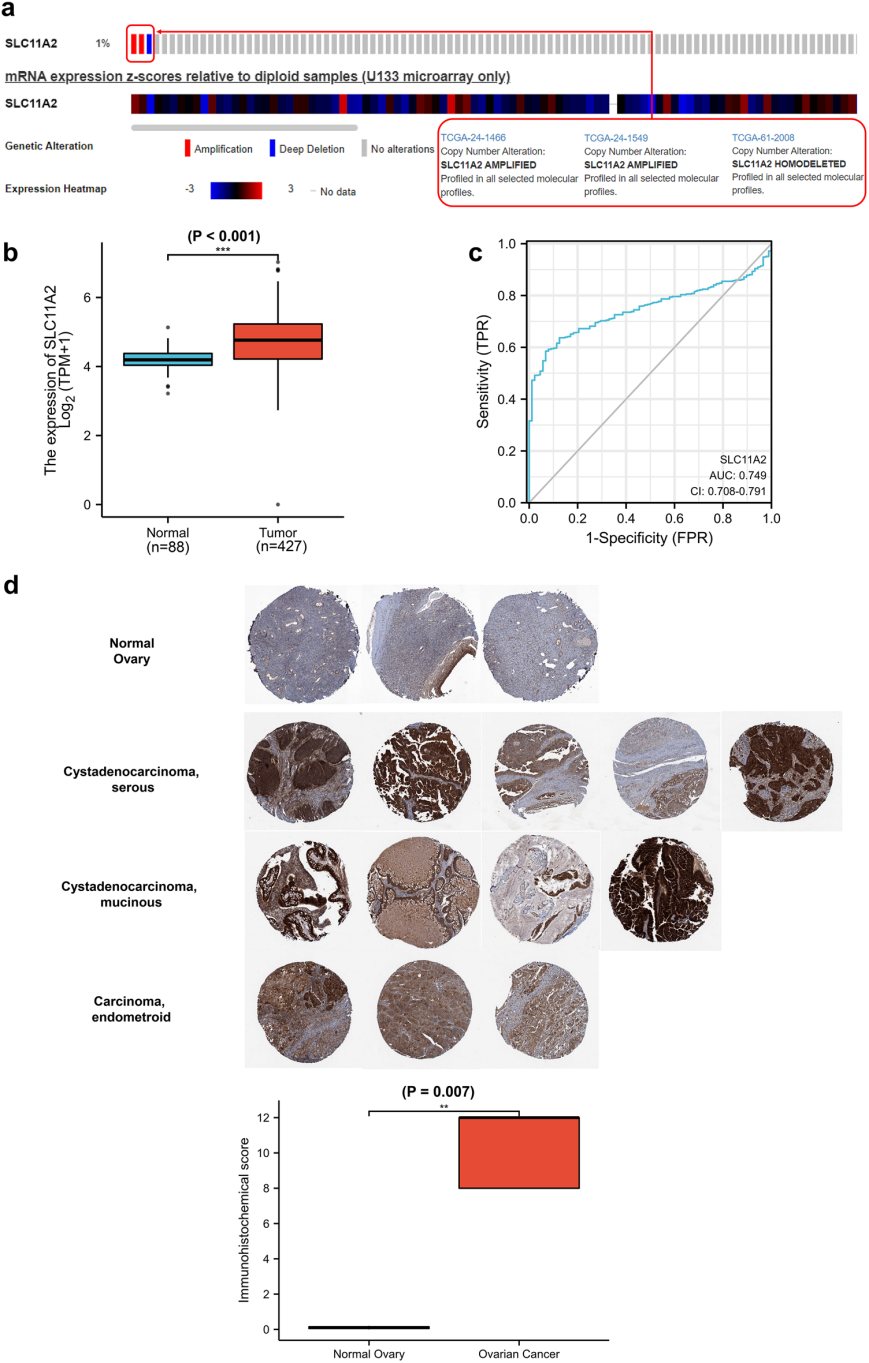
SLC11A2 mRNA and protein expression in ovarian cancer. (**a**) The genomic change rate of the SLC11A2 gene in ovarian serous. (**b**) SLC11A2 mRNA in ovarian serous carcinoma, combined from TCGA and GTEx. Statistical methods: Wilcoxon rank sum test. (Statistical P-values represented by asterisks: *P < 0.05; **P < 0.01; ***P < 0.001; the below is the same). (**c**) ROC (Receiver operating characteristic) curve of SLC11A2 mRNA for ovarian cancer diagnosis. (**d**) IHC (Immunohistochemical) of SLC11A2 protein in normal ovarian tissue and ovarian serous carcinoma tissue (The Human Protein Atlas; https://www.proteinatlas.org/).

The CbioPortal database found that the genomic change rate of the SLC11A2 gene in ovarian serous carcinoma was 1%. It indicated that as a vital molecule of iron transport, SLC11A2 was relatively conserved in the genome. The following heat map shows the expression level of SLC11A2 in ovarian cancer (Fig. 2a). Combined analysis of ovarian cancer tissues from the TCGA database and normal ovarian tissues from the GTEx database revealed elevated levels of SLC11A2 mRNA in ovarian serous carcinoma (Fig. 2b, Supplementary Table S2). Using tissue SLC11A2 mRNA transcript levels as a predictor of ovarian cancer, the area under the curve AUC was 0.749, and the confidence interval CI was 0.708–0.791 (Fig. 2c). That is, the accuracy of determining benign and malignant was 74.9% using the SLC11A2 mRNA transcript level of resected ovarian tissue as a marker. This shows its potential as a biomarker for ovarian cancer.

The Human Protein Atlas database immunohistochemical staining showed that SLC11A2 protein was highly expressed in ovarian serous carcinoma and not expressed in normal ovarian tissue. The trend of protein expression and mRNA transcription levels in ovarian cancer was consistent. The three main pathological classifications of epithelial ovarian cancer are shown in the figure, and statistical analysis shows that the expression of the SLC11A2 protein is significantly increased in ovarian cancer. (Fig. 2d, P < 0.001).

### Ovarian cancer with high expression of SLC11A2 has a worse prognosis

**Figure 3:**
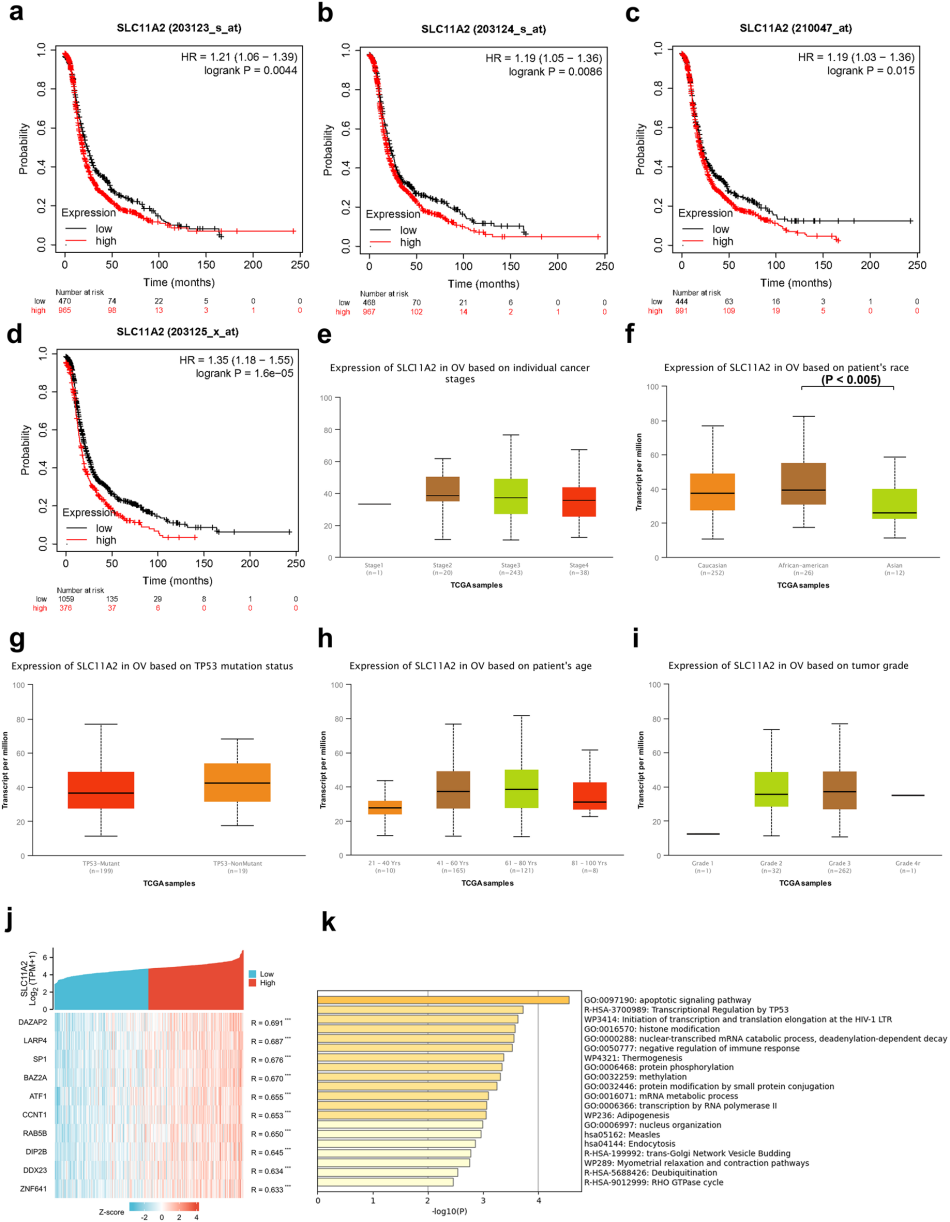
Relationship between SLC11A2 and ovarian cancer prognosis; Cell signaling pathway enrichment analysis. (**a**–**d**) Kaplan-Mayer PFS curves over high and low SLC11A2 mRNA expression. (**f**–**i**) Correlation between SLC11A2 and clinical features of ovarian cancer. (**j**) Top 10 positively correlated genes with SLC11A2 expression. (**k**) SLC11A2 co-expressed gene enrichment analysis in ovarian cancer. (Metascape tool).

We used the Kaplan-Mayer plotter web tool to obtain the mRNA expression of SLC11A2 in ovarian cancer corresponding to 4 different probe matrices. The 4 matrices are: 203123_s_at (SLC11A2), 203124_s_at (SLC11A2), 203125_x_at (SLC11A2), 210047_at (SLC11A2), and the cases were consistent with each probe (n = 1435). SLC11A2 transcript levels were divided into a high-expression group (red) and low expression (black), and progression-free survival (PFS) was proxies for prognosis. The X-axis is PFS time and the Y-axis is survival probability. All four arrays showed that ovarian cancer patients with high SLC11A2 expression had shorter progression-free survival (PFS) than those with low expression (P = 0.0044, 0.0086, 0.015, 0.000016; HR = 1.21, 1.19, 1.19, 1.35) and the median survival time was also significantly different: 1.73–4.78 months (Fig. 3a–d; Supplementary Table S3).

The relationship between clinical features and expression was obtained from the UALCAN database. Correlation of ovarian cancer SLC11A2 mRNA with FIGO stage, ethnicity, TP53 mutation status, age, and grade of pathological differentiation (Fig. 3e–i) showed that: SLC11A2 was only significantly different in ethnicity, Asian ovarian cancer Patients had low SLC11A2 mRNA expression. This means that local research on this molecule is necessary.

### SLC11A2 expression is associated with apoptosis in ovarian cancer

Heatmaps were drawn using the top 10 genes that were positively correlated with SLC11A2 expression in ovarian cystadenocarcinoma (Fig. 3j, Supplementary Table S4). These molecules can provide clues for subsequent studies on the protein interaction of SLC11A2. Gene ontology and signal pathway analysis indicated that a high proportion of co-expressed molecules functioned in the apoptotic signaling pathway, negative regulation of immune response, and transcriptional regulation by TP53 (Fig. 3k).

### Knockdown of SLC11A2 reduced colony-forming ability of ovarian cancer cells

**Figure 4:**
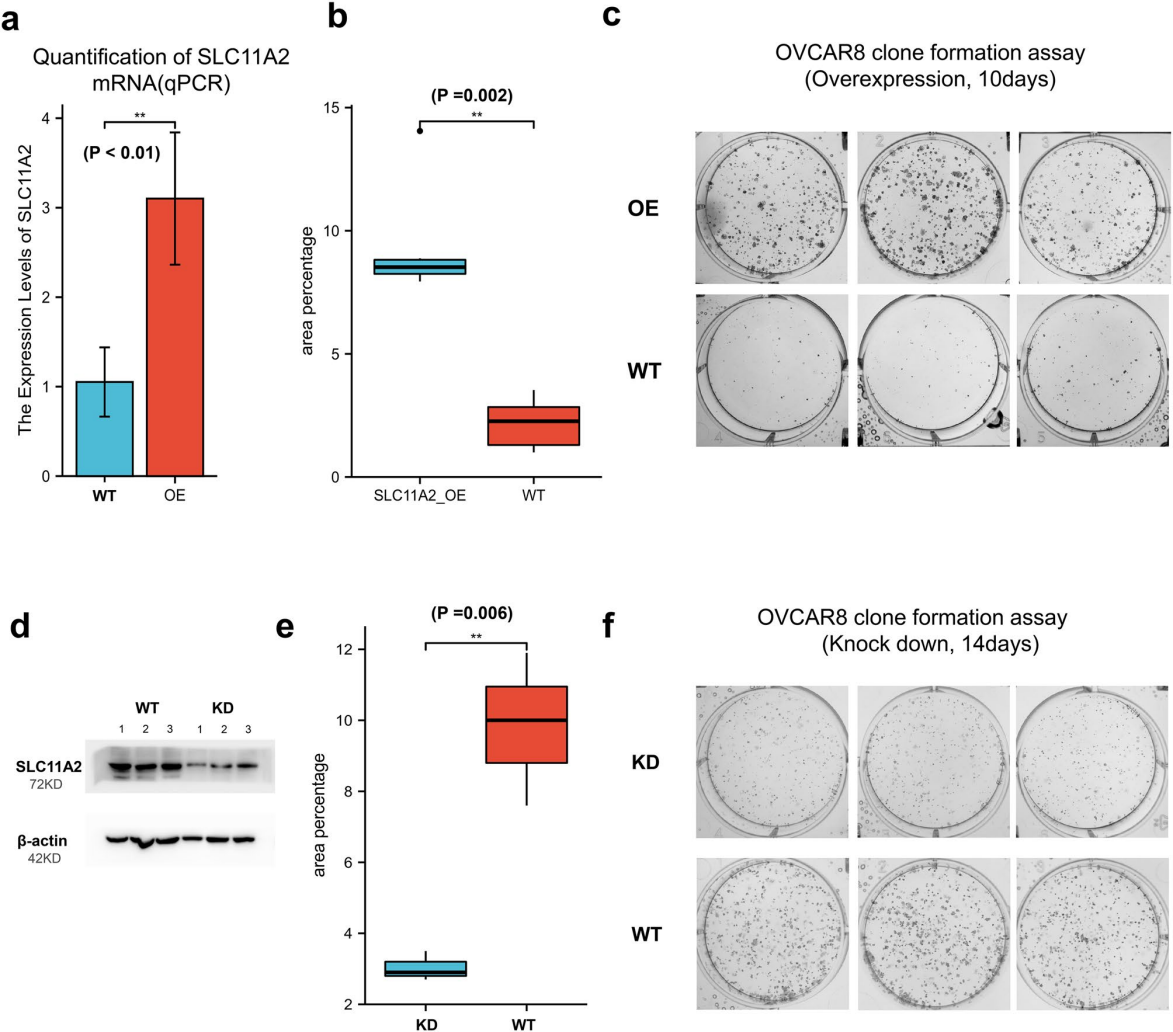
The effect on the colony formation of ovarian cancer cell lines. (**a**) Detection of overexpression efficiency: Wild type (WT) vs Overexpressed (OE). (**b**) Quantification of colony formation (OE vs WT). Statistical methods: independent samples t-test. (**c**) Images of colony formation. (Overexpression). (**d**) Verification of knock-down efficiency by western blot, SLC11A2, and β-actin. Blots were cropped prior to incubation with primary antibody hybridization. Original blots are presented in Supplementary Fig. S1. (**e**) Quantification of colony formation, Wild type (WT) vs knockdown (KD). Statistical methods: independent samples t-test. (**f**) Images of colony formation (knockdown).

Colony-forming results showed that the survival rate and replication efficiency of cells were significantly improved after SLC11A2 overexpression (Fig. 4a–c; P = 0.002). To further demonstrate, we used siRNA to specifically inhibit the translation of SLC11A2 and compared it to negative si-RNA control. The knockdown efficiency was verified by Western blot (Fig. 4d; original blots are presented in Supplementary Fig. S1). The results showed that the knockdown of SLC11A2 significantly improved the survival rate and replication efficiency of ovarian cancer cells (Fig. 4e,f; P = 0.006). Cell clone formation experiments showed that the expression level of SLC11A2 was positively correlated with the survival rate and replication rate.

### Knockdown of SLC11A2 inhibited ovarian cancer proliferation and migration

**Figure 5:**
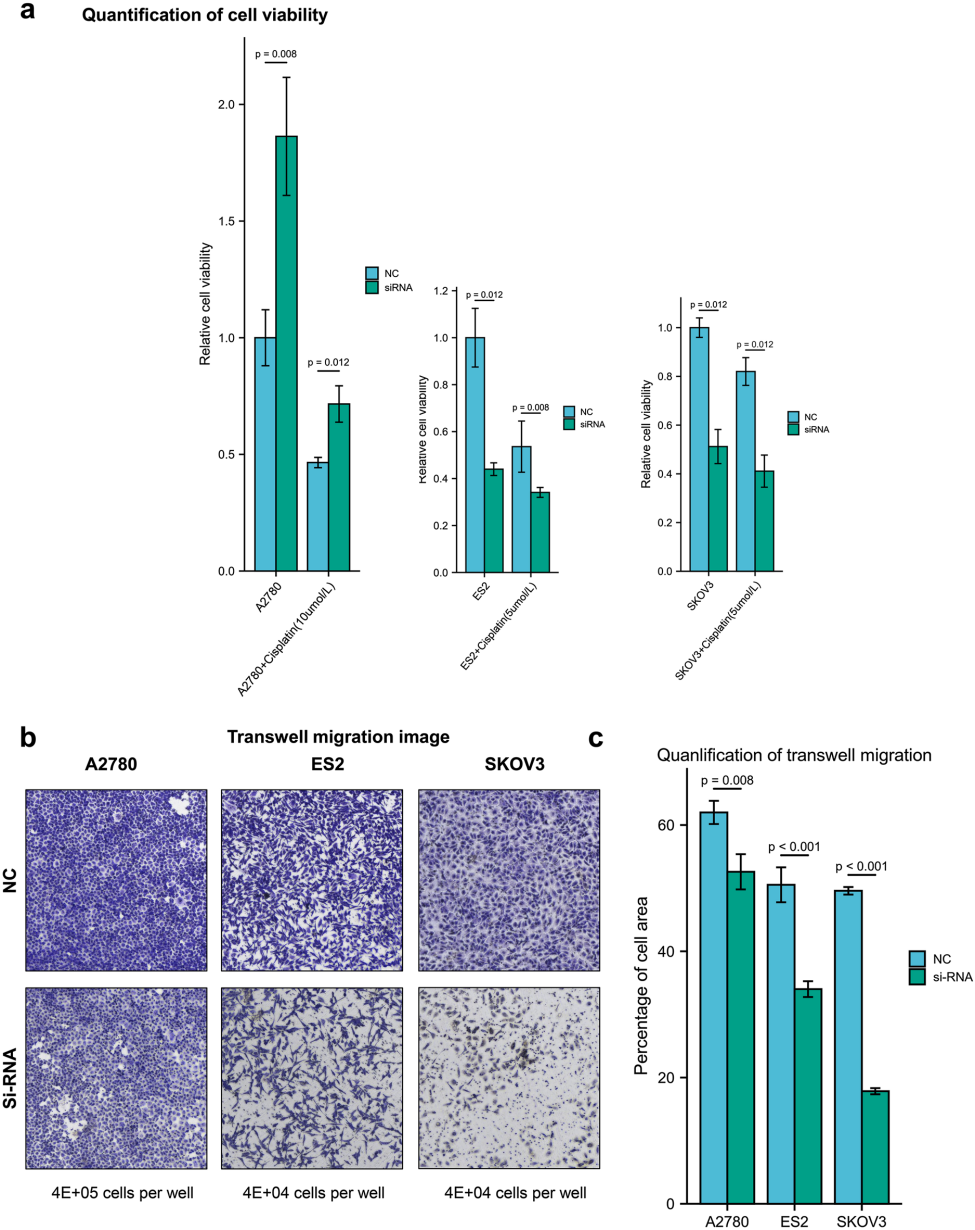
Effects of SLC11A2 knockdown on cell viability and migration. (**a**) Cell viability treated with cisplatin via CCK8 assay. Statistical methods: independent samples t-test. (**b**) Image of Transwell migration assay. (NC vs siRNA). (**c**) Quantification of Transwell migration assay. Statistical methods: independent samples t-test.

The CCK8 cell viability assay of A2780, ES2, and SKOV3 showed that the viability of A2780 cells increased after knocking down SLC11A2; however, the viability of ES2 and SKOV3 cells decreased. This trend was not affected by the addition of cisplatin to the culture medium (Fig. 5a).

Transwell cell migration assays showed that the expression level of SLC11A2 was positively correlated with the migration ability of ovarian cancer cell lines (Fig. 5b,c). Knockdown of SLC11A2 could significantly inhibit the migration ability of ovarian cancer cells.

### Knockdown of SLC11A2 significantly increased ovarian cancer sensitivity to cisplatin

**Figure 6:**
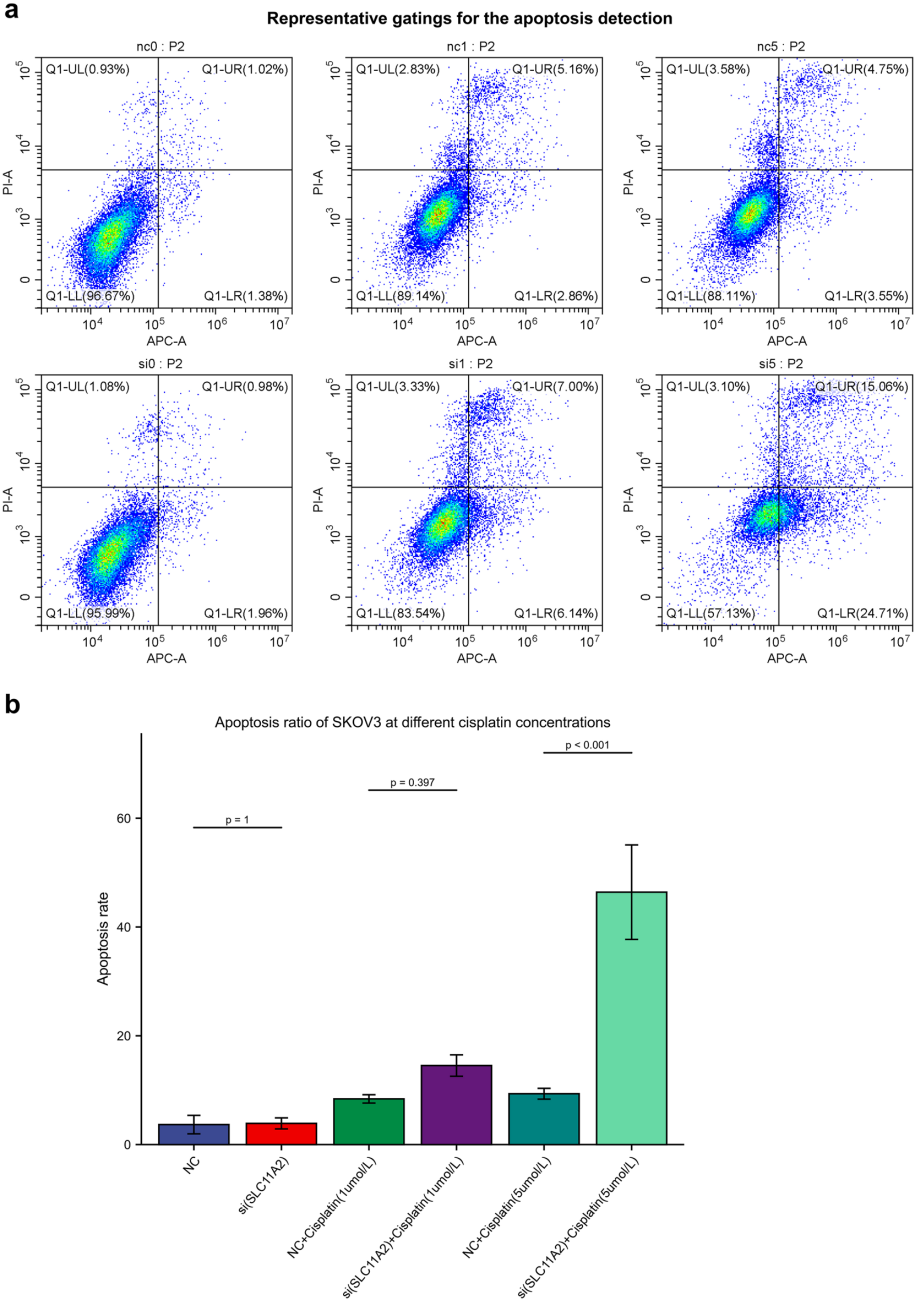
SLC11A2 knockdown increased cisplatin sensitivity in SKOV3 cell line. (**a**) Flow cytometry plots of skov3 cells apoptosis. The total amount of apoptosis was early apoptosis plus late apoptosis (right two quadrants). This is a representative plot of 3 independent replicate experiments. (**b**) Bar graph of percentage apoptosis. Statistical methods: Welch's t-test.

In the cell viability experiments with the knockdown of SLC11A2, the SKOV3 cell line showed the greatest decrease in viability, so we selected the SKOV3 cell line for further work. We counted the apoptosis ratio of SKOV3 cells induced by cisplatin, and the results showed that the knockdown of SLC11A2 significantly increased the proportion of ovarian cancer apoptosis induced by a certain concentration of cisplatin (Fig. 6a,b).

### Tissue distribution characteristics and prognostic relevance of SLC11A2 protein

**Figure 7:**
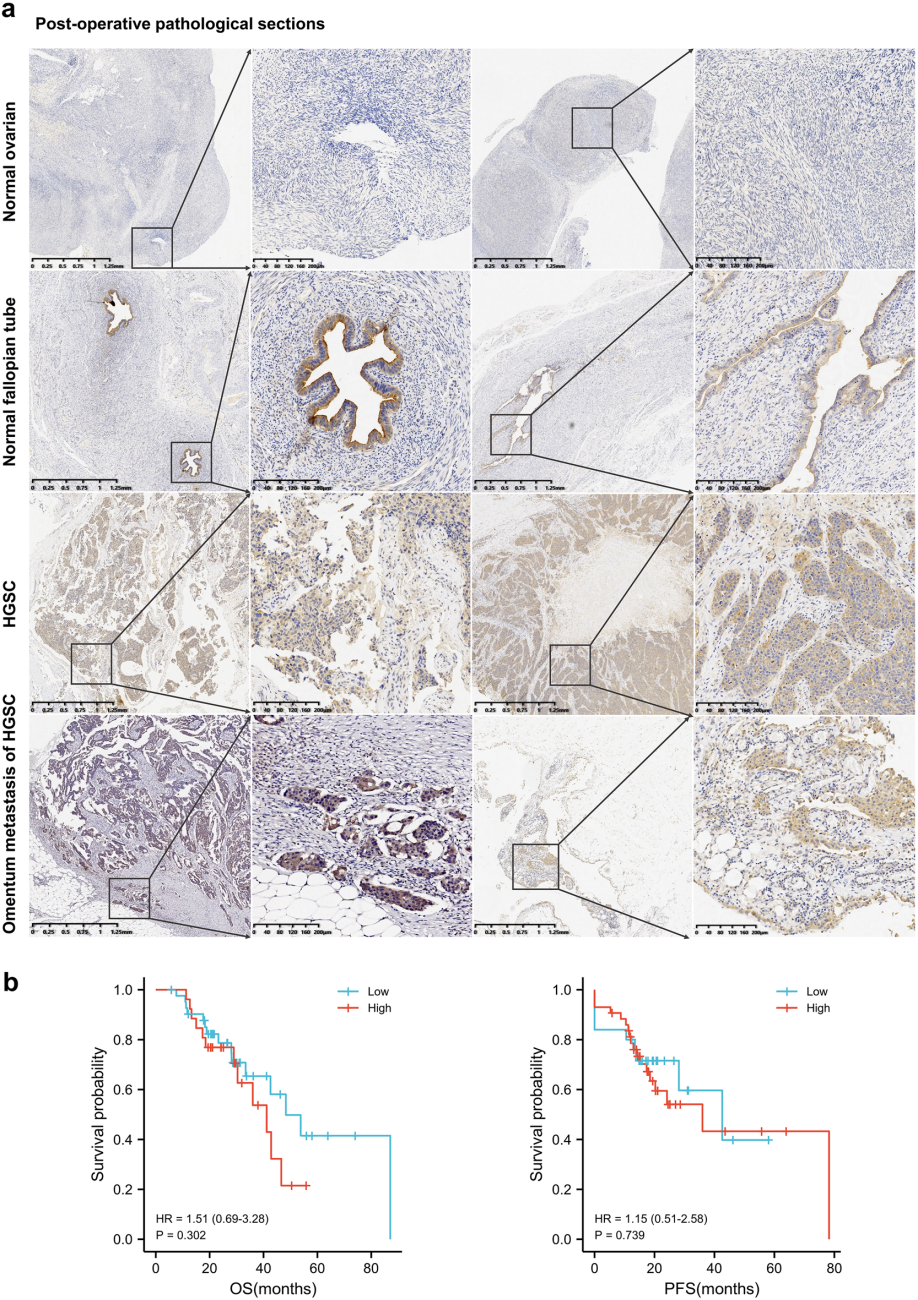
SLC11A2 immunohistochemical analysis. (**a**) Distribution of SLC11A2 protein in 4 tissue types: pathological sections of the normal ovary, normal fallopian tube, ovarian high-grade serous carcinoma, and ovarian carcinoma with omental metastases. Two samples of each tissue type were selected, each sample with two magnifications. (**b**) The relationship between the expression of SLC11A2 protein in primary ovarian cancer and prognosis (IHC, n = 68). Statistical methods: Cox regression analysis.

Immunohistochemical results showed that SLC11A2 protein was strongly positive in ovarian serous carcinoma primary foci, omentum metastases, and normal fallopian tube mucosa. Completely negative in other parts of normal ovaries and fallopian tubes. This is somewhat different from the mRNA transcript level In normal ovaries, SLC11A2mRNA has a certain level of transcription, but immunohistochemistry showed that the protein was not distributed in normal ovaries. Its distribution in the fallopian tubes is also clearly specific: it is only highly expressed in the fallopian tube mucosa. All specimens are consistent; 2 of which are shown separately in the figure. This figure provides a larger view and cross-sectional expression of the fallopian tube that differs from the tissue microarray database (Fig. 7a).

The retrospective immunohistochemical study showed that patients with high SLC11A2 expression in the primary tumor had shorter OS and PFS, but this trend was not statistically significant (OS, P = 0.302, HR = 1.51; PFS, P = 0.739, HR = 1.15). From the curve and HR value, the patients with low expression of SLC11A2 protein have more obvious advantages in OS than PFS (Fig. 7b). The lack of statistical significance may be related to the small sample size. Clinical baseline information for this study cohort is in Supplementary Table S5.

### SLC11A2 is elevated in ovarian cancer blood

**Figure 8:**
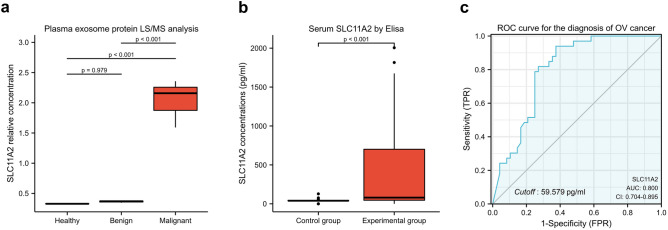
Expression of SLC11A2 in the serum of ovarian cancer patients. (**a**) Detection of SLC11A2 protein concentration in plasma exosomes of ovarian cancer patients using liquid chromatography-mass spectrometry. Statistical Methods: one-way ANOVA test. (**b**) The concentration of SLC11A2 in serum was detected with the Elisa kit. Statistical Methods: one-way ANOVA test. (**c**) Receiver operating characteristic (ROC) curve of SLC11A2 in serum to detect ovarian cancer. The best cutoff value and area under the curve (AUC) are marked in the figure.

Only 8 of the 27 plasma exosome samples were detected and quantified at SLC11A2 concentrations (Fig. 8a). The relative concentration value of SLC11A2 protein in plasma exosomes of ovarian cancer patients was significantly higher than that of healthy and benign ovarian disease groups. Considering the detection rate and cost, we used the Elisa kit to detect a large number of blood samples. Clinical baseline information for this study cohort is in Supplementary Table S6.

ELISA results showed that serum SLC11A2 concentrations in patients with a high burden of ovarian cancer (untreated and relapsed) were significantly higher than in women without an ovarian cancer burden (Fig. 8b) The receiver operating curve showed that the best cut-off value for distinguishing ovarian cancer from non-ovarian cancer was: 59.579 pg/ml, and the area under the curve (AUC) was 0.8, which means that SLC11A2 has the potential to act as a serological marker for ovarian cancer (Fig. 8c).

## Discussion

Ovarian cancer remains the malignant tumor with the highest mortality rate among gynecological tumors^18^. Because the ovary is located in the deep pelvis^19^, ovarian cancer has no obvious symptoms, and most of the diagnosed patients are in the advanced stage (FIGO III-IV stage)^20^, and the ovarian is an abdominal organ with a rapid disease progression. According to statistics^21^, the 5-year survival rate of ovarian cancer is 76–93% for stage I, 60–74% for stage II, 23–41% for stage III, and 11% for stage IV. Early diagnosis and improvements in treatment options are the keys to curing ovarian cancer. The former relies on fluid examination and imaging studies. The latter relies on the innovation of anti-tumor drugs, because the improvement of the cure rate of surgery is close to the limit, at the Same Time most ovarian cancers are not sensitive to radiotherapy. Our research aims to find new molecular diagnostic markers and therapeutic targets for ovarian cancer. SLC11A2 is a protein responsible for iron transport in cells^22^. Most of the current research focuses on the role of this molecule in the iron-overloaded hematopoietic system. Until recent years, some cancer-related studies on this molecule have been reported. The cancer-promoting role of SLC11A2 has been demonstrated in colon and breast cancer, which attracted our attention^23^.

Survival analysis showed that the high SLC11A2 mRNA expression group had poor prognosis than the low expression group in ovarian cancer patients, and the results of the 4 arrays were similar. The PFS risk rate (HR) ranges from 1.19 to 1.35. Although the HR is not high as it looks, considering that most of the ovarian cancer nests are diffusely expressed in the immunohistochemical results and normal ovaries are completely negative, indicated that SLC11A2 is highly expressed in the vast majority of ovarian cancers. In the case of such high expression, the prognostic effect of subtle differences in expression levels will not be obvious enough.

We calculated the difference in MST (median survival time) corresponding to the 4 chips, which were 4.78 months, 3.57 months, 4.44 months, and 1.73 months, respectively. In comparison, even the current PARP inhibitor maintenance therapy for all ovarian cancers prolongs median survival by only 12.9 months, reduces the risk of death by 26%, and improves 5-year survival by 9%^24^ (SOLO2)^25^. In this way, the benefit of SLC11A2 as a therapeutic target is considerable.

Subgroup analyses of clinical characteristics revealed some trends, however, only P values in racial expression were significantly different. This indicates that the racial differential expression between Asians and other races is more pronounced. To this end, we subsequently collected blood and tissue samples to obtain real data from Asian patients.

In our CCK8 cell proliferation assay, the A2780 cell line and other cell lines responded oppositely to the modulation of SLC11A2. This is similar to the previous study of humans in breast cancer^26^, in which the invasive breast cancer cell line MB231 and the non-invasive breast cancer MCF-7 were cultured separately with deferoxamine (DFO) to reduce the iron concentration of the medium. Increased iron concentration in MB231 cytoplasm and mitochondrial resulted in enhanced cell proliferation. On the other hand, reduced iron concentration in cytoplasm and mitochondria decreased MCF-7 cell line viability. This shows that different cancer cells have different responses to iron regulation, which is also important that needs to be considered when we take iron regulation-related molecules as cancer therapeutic targets.

Platinum alkylating agents-based chemotherapy is the first-line chemotherapy for ovarian cancer, so we used cisplatin (platinum chemotherapy drugs) as an inducer to detect the effect of SLC11A2 knockdown on ovarian cancer cells. The apoptosis pathway ranked first in the GO analysis above, so we first detected the effect of SLC11A2 knockdown and cisplatin in inhibiting cell proliferation, and then selected the apoptosis rate as the detection target. Clinically, the side effects and drug resistance of platinum-based chemotherapy are important factors affecting efficacy and recurrence. Knockdown of SLC11A2 has a significant promoting effect on platinum-induced cancer apoptosis, which makes it possible to reduce the dose of platinum in clinical practice and reduce side effects and drug resistance recurrence.

The immunohistochemical staining results were unexpected and exciting. According to the results of transcriptome sequencing, the transcript of SLC11A2 in normal ovarian tissue was not low. However, the SLC11A2 protein was not detected in the immunohistochemical staining of all normal ovaries. The normal fallopian tube mucosa was strongly positive, while the muscular, stroma, and serosa were negative. The mucosal layer is composed of a single layer of tall columnar cells and plays an important role in ovulation and fertilization. We have not yet been able to tell which type of mucosal layer cells express such high levels of SLC11A2. This expression pattern corroborates the fallopian tube origin theory proposed by the academic community in recent years for high-grade serous carcinoma of ovarian cancer^27, 28^. The immunohistochemical prognostic curves of advanced ovarian cancer show that patients with high SLC11A2 expression have shorter OS. Although the sample size was limited, the trend of the curve was quite obvious.

At the same time, we propose a hypothesis: SLC11A2 acts as an iron transporter protein in the fallopian tube mucosa to maintain a low iron concentration environment in the fallopian tube lumen. Menstrual blood enters the lumen of the fallopian tubes in women of childbearing age, and the menstrual blood is cleared by macrophages. Without transport means, iron engulfed by intratubular macrophages would form a high concentration of iron in the tubal fluid and deposit on the tube wall, which would interfere with the ovulation and fertilization process. Studies^29, 30^ have shown that the concentration of iron ions in the lumen of the fallopian tube affects both macrophages and sperm. SL11A2 is the molecule that transports iron ions out of the fallopian tube to maintain a normal tubal fluid environment to help ovulation and fertilization. In addition to the promise of treating infertility, SLC11A2 could in the future be applied by pathologists to determine the source of ovarian tumors.

In the absence of feasible preventive measures for ovarian cancer, early diagnosis is an important way to reduce mortality. Exosomes have been a research hotspot in the past decade. We extracted plasma exosomes from 27 paired patients for Liquid Chromatography Mass Spectrometry (LC–MS). Although only 8 cases were successfully quantified for SLC11A2 protein, it was enough to show significant differences. The cost of exosome extraction and HPLC–MS remains high in a short period, which limits the clinical application of this technology. At the same time, to answer the problem of the low detection rate of mass spectrometry, we tried the Elisa kit with an avidin amplification effect. Since there is no literature showing that anyone has used the kit of this method and test kits to detect SLC11A2, the stability of the kit also needs more experiments to prove. The available evidence shows that even if it cannot be an independent diagnostic indicator, it is hopeful to become a combined diagnostic indicator to help improve the overall detection rate.

This study showed that high SLC11A2 mRNA and protein expression in ovarian cancer tissues tends toward a worse prognosis. Knockdown of SLC11A2 increased cisplatin-induced apoptosis in ovarian cancer cells. This study suggests that SLC11A2 may be a potential therapeutic target and combined diagnostic biomarker for ovarian cancer.

## Supplementary Information

Supplementary Information 1.

Supplementary Information 2.

## Data Availability

The data that support the findings of this study are available from the corresponding author upon reasonable request.

## Abbreviations

OV: Ovarian cancer
SLC11A2: Solute carrier family 11-member 2
TCGA: The Cancer Genome Atlas
GTEx: Genotype-tissue expression
GEPIA: Gene expression profiling interactive analysis
MST: Median survival time
HGSC: High grade serous ovarian cancer
AUC: Area under curve
CI: Confidence interval
PARP: Poly (ADP-ribose) polymerase
GENT: Gene expression in normal and tumor tissue
FIGO: International federation of gynecology and obstetrics
HR: Hazard ratio
GeneMANIA: Gene multiple association network integration algorithm
GSEA: Gene set enrichment analysis
PDL1: Programmed death ligand 1
NC: Negative control
OE: Overexpressed
WT: Wild type
KD: Knock down
CCK8: Cell counting Kit-8
Fig.: Figure
ROC: Receiver operating characteristic
TF: Transferrin
