# Supplementary material for: SLC11A2: a promising biomarker and therapeutic target in ovarian cancer
Source: Sci Rep. 2023 Jan 20;13:1132. doi: 10.1038/s41598-022-26789-5 (PMC9860018; doi:10.1038/s41598-022-26789-5)
Supplement: Supplementary file 1 — Supplementary Information 1. [file 41598_2022_26789_MOESM1_ESM.docx]

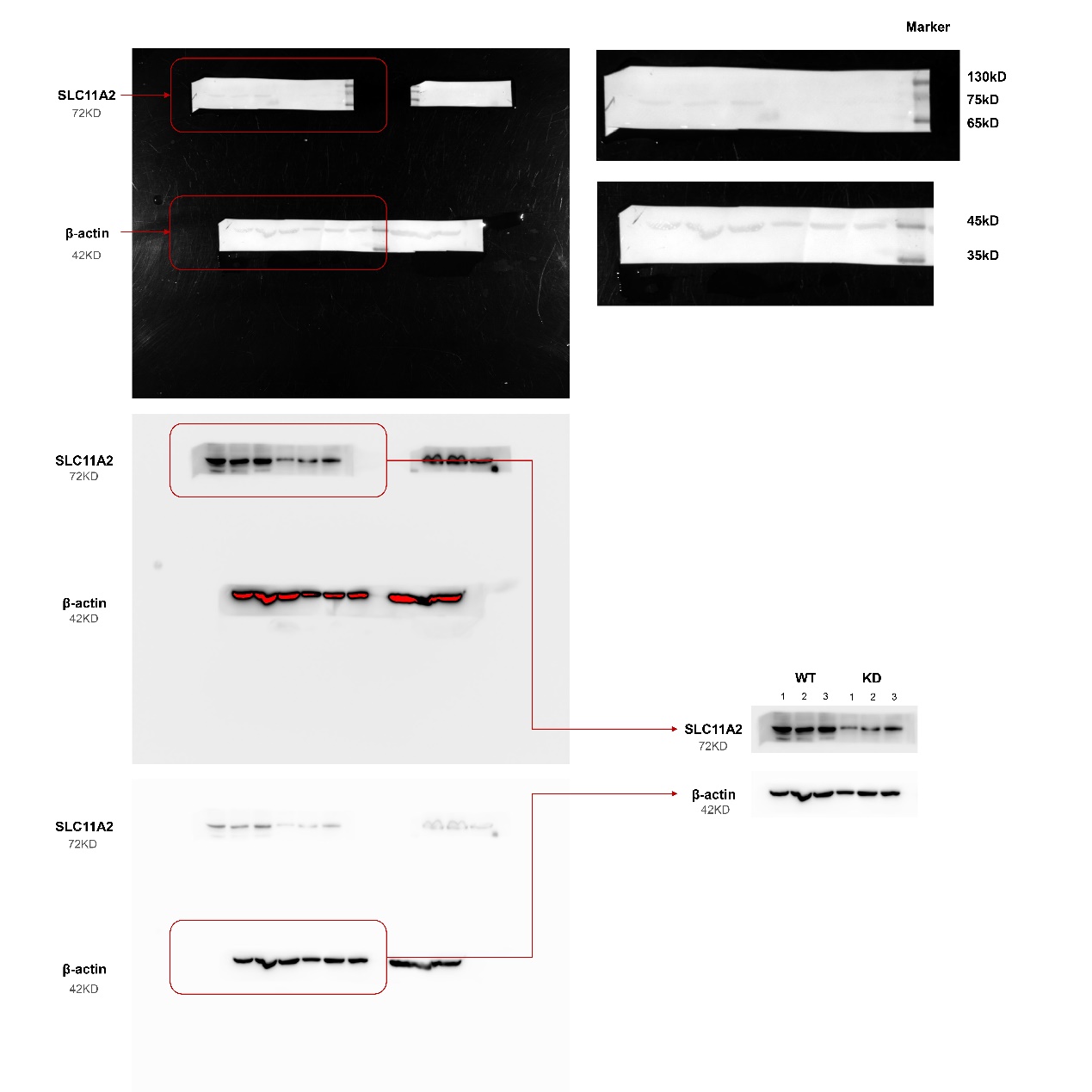
**Supplementary Figure 1. Original Western blot blots of Figure 4d**

Western blots were cropped prior to incubation with primary antibody hybridization. The top left corner shows the position of the bands when exposed to Western blot, and the top right corner shows the molecular weight of the marker bands. The middle and bottom left panels show the strips at two different exposure times. The underexposed bands were extracted separately and combined to form the Fig4d plot on the right.
