# Supplementary material for: SLC11A2: a promising biomarker and therapeutic target in ovarian cancer
Source: Sci Rep. 2023 Jan 20;13:1132. doi: 10.1038/s41598-022-26789-5 (PMC9860018; doi:10.1038/s41598-022-26789-5)
Supplement: Supplementary file 2 — Supplementary Information 2. [file 41598_2022_26789_MOESM2_ESM.docx]

**Supplementary Table 1-a.**

**Differential expression statistics corresponding to each cancer type [GPL570 platform]**

| **tissue** | **P-value** | **Log2FC** |
| --- | --- | --- |
| All | <0.001 | -0.052 |
| Adipose | 0.052 | -0.29 |
| Adrenal Gland | <0.001 | 0.578 |
| Bladder | 0.015 | 0.268 |
| Blood | <0.001 | 0.096 |
| Bone | 0.078 | 0.417 |
| Brain | <0.001 | -0.202 |
| Breast | <0.001 | 0.168 |
| Cervix | 0.304 | -0.163 |
| Colon | <0.001 | 0.876 |
| Endometrium | 0.003 | 0.301 |
| Esophagus | 0.348 | 0.13 |
| Eye | NA | -1.667 |
| Gallbladder | 0.677 | -0.128 |
| Head and Neck | <0.001 | -1.132 |
| Kidney | <0.001 | -0.299 |
| Liver | <0.001 | 0.14 |
| Lung | <0.001 | 0.117 |
| Lymph Node | NA | 1.097 |
| Muscle | 0.563 | -0.13 |
| Oral | 0.789 | -0.055 |
| Ovary | <0.001 | 0.207 |
| Pancreas | 0.359 | 0.07 |
| Pharynx | 0.896 | 0.095 |
| Placenta | NA | NaN |
| Prostate | 0.049 | 0.293 |
| Skin | <0.001 | -0.291 |
| Small Intestine | 0.299 | -0.697 |
| Spleen | 0.638 | 0.09 |
| Stomach | <0.001 | 0.451 |
| Teeth | 0.27 | -0.315 |
| Testis | 0.076 | -0.301 |
| Thyroid | <0.001 | -0.163 |
| Tongue | 0.023 | -0.496 |
| Uterus | 0.767 | 0.04 |
| Vagina | 0.585 | 0.218 |
| Vulva | 0.046 | -0.312 |

**Supplementary Table 1-b.**

**Differential expression statistics corresponding to each cancer type [GPL96 platform]**

| **tissue** | **P-value** | **Log2FC** |
| --- | --- | --- |
| All | 0.051 | 0.025 |
| Adrenal Gland | 0.374 | -0.102 |
| Bladder | 0.946 | 0.020 |
| Blood | <0.001 | 0.400 |
| Bone M arrow | <0.001 | -0.285 |
| Brain | <0.001 | 0.170 |
| Breast | <0.001 | -0.245 |
| Cartilage | 0.319 | -1.883 |
| Cervix | 0.108 | -0.255 |
| Colon | <0.001 | 0.773 |
| Esophagus | <0.001 | -0.312 |
| Heart | <0.001 | -0.771 |
| Immune System | <0.001 | -0.401 |
| Joint | 0.184 | 0.140 |
| Kidney | <0.001 | -0.156 |
| Larynx | 0.589 | -0.205 |
| Liver | 0.004 | -0.178 |
| Lung | <0.001 | 0.161 |
| Muscle | 0.857 | 0.137 |
| Ovary | <0.001 | 0.399 |
| Pancreas | <0.001 | -0.933 |
| Pharynx | 0.753 | -0.156 |
| Prostate | 0.315 | -0.071 |
| Skin | 0.003 | -0.214 |
| Small Intestine | NA | NA |
| Soft Tissue | 0.006 | 0.313 |
| Stomach | <0.001 | 0.194 |
| Testis | 0.034 | 0.227 |
| Thyroid | 0.008 | 0.230 |
| Tongue | 0.033 | 0.686 |
| Urothelium | 0.738 | -0.034 |
| Uterus | 0.887 | 0.017 |

**Supplementary Table 2.**

**Expression of SLC11A2 mRNA in ovarian cancer and normal ovarian tissue**

| Group | Normal | Tumor |
| --- | --- | --- |
| Number | 88 | 427 |
| least value | 3.217 | 0 |
| Maximum value | 5.133 | 7.025 |
| Median (Median) | 4.193 | 4.764 |
| Quartile interval (IQR) | 0.34 | 1.015 |
| Lower quartile | 4.038 | 4.217 |
| Upper quartile | 4.378 | 5.232 |
| Mean value (Mean) | 4.194 | 4.713 |
| Standard deviation (SD) | 0.307 | 0.766 |
| Standard error (SE) | 0.033 | 0.037 |

**Supplementary Table 3.**

**Median survival time of 4 probe set**

| **probe of arrays** | **MST (median survival time)** | | |
| --- | --- | --- | --- |
|  | **Low expression cohort (months)** | **High expression cohort (months)** | **Difference (months)** |
| 203123_s_at (SLC11A2) | 23.57 | 18.79 | 4.78 |
| 203124_s_at (SLC11A2) | 22.5 | 18.93 | 3.57 |
| 203125_x_at (SLC11A2) | 21.47 | 17.03 | 4.44 |
| 210047_at (SLC11A2) | 21 | 19.27 | 1.73 |

**Supplementary Table 4**

**Top 10 genes positively correlated with SLC11A2 in Ovarian Cancer (TCGA)**

| **Target molecule** | **Other molecules** | **Correlation coefficient (Pearson)** | **P-value (Pearson)** | **Correlation coefficient (Spearman)** | **P-value (Spearman)** |
| --- | --- | --- | --- | --- | --- |
| SLC11A2 | DAZAP2 | 0.691 | <0.001 | 0.698 | <0.001 |
| SLC11A2 | LARP4 | 0.687 | <0.001 | 0.706 | <0.001 |
| SLC11A2 | SP1 | 0.676 | <0.001 | 0.687 | <0.001 |
| SLC11A2 | BAZ2A | 0.670 | <0.001 | 0.693 | <0.001 |
| SLC11A2 | ATF1 | 0.655 | <0.001 | 0.655 | <0.001 |
| SLC11A2 | CCNT1 | 0.653 | <0.001 | 0.671 | <0.001 |
| SLC11A2 | RAB5B | 0.650 | <0.001 | 0.690 | <0.001 |
| SLC11A2 | DIP2B | 0.645 | <0.001 | 0.667 | <0.001 |
| SLC11A2 | DDX23 | 0.634 | <0.001 | 0.652 | <0.001 |
| SLC11A2 | ZNF641 | 0.633 | <0.001 | 0.654 | <0.001 |

**Supplementary Table 5**

**Grouping of Immunohistochemical analysis (OS)**

| **Group** | **Numbers** | **MST(months)** |
| --- | --- | --- |
| **Low** | 42 | 48.3 |
| **High** | 26 | 41.2 |

**Grouping of Immunohistochemical analysis (PFS)**

| **Group** | **Numbers** | **MST(months)** |
| --- | --- | --- |
| **Low** | 40 | 42.6 |
| **High** | 28 | 36 |

**Baseline information of the IHC cohort of ovarian cancer patients**

| **Items** | **Numbers** |
| --- | --- |
| **Ages** | 56.12±16.58 years |
| **FIGO stage** |  |
| III | 61.8%, (n=42) |
| IV | 38.2%, (n=26) |
| **Maintenance therapy with PARPi** | 22.1%, (n=15) |
| **Outcomes** |  |
| Survival | 60.3%, (n=41) |
| Death | 39.7%, (n=27) |
| **Immunohistochemistry score** |  |
| **Dyeing intensity** |  |
| Negative | 1.47%, (n=1) |
| Weakly positive | 14.7%, (n=10) |
| Positive | 36.8%, (n=25) |
| Strongly positive | 47.1%, (n=32) |
| **Percentage of positive cells in cancer foci** |  |
| 0%-25% | 1.47%, (n=1) |
| 26%-50% | 13.2%, (n=9) |
| 51%-75% | 52.9%, (n=36) |
| 75%-100% | 32.4%, (n=22) |

**Supplementary Table 6**

**Grouping of serum Elisa assays**

| **Items** | **Experimental group** | **Control group** |
| --- | --- | --- |
| **Gender** |  |  |
| Female | 48 | 33 |
| Male | 0 | 0 |
| **Age** | 53.61±15.93**(years)** | 49.12±13.94**(years)** |
| **Diagnosis** |  |  |
| Health volunteers | 0 | 10 |
| Benign ovarian lesions | 0 | 2 |
| Borderline ovarian tumor | 0 | 4 |
| Post-operative ovarian cancer | 0 | 9 |
| Colon cancer | 0 | 8 |
| Untreated ovarian cancer | 44 | 0 |
| Recurrent ovarian cancer | 4 | 0 |
